# Greek Graviera Cheese Assessment through Elemental Metabolomics—Implications for Authentication, Safety and Nutrition

**DOI:** 10.3390/molecules24040670

**Published:** 2019-02-14

**Authors:** Georgios Danezis, Charis Theodorou, Theofilos Massouras, Evangelos Zoidis, Ioannis Hadjigeorgiou, Constantinos A. Georgiou

**Affiliations:** 1 Chemistry Laboratory, Department of Food Science and Human Nutrition, Agricultural University of Athens, 11855 Athens, Greece; gdanezis@aua.gr (G.D.); cag@aua.gr (C.A.G.); 2 Department of Nutritional Physiology and Feeding, Faculty of Animal Science and Aquaculture, Agricultural University of Athens, 11855 Athens, Greece; charistheodoroy@gmail.com (C.T.); ezoidis@aua.gr (E.Z.); ihadjig@aua.gr (I.H.); 3 Dairy Science and Technology Laboratory, Department of Food Science and Human Nutrition, Agricultural University of Athens, 11855 Athens, Greece; theomas@aua.gr

**Keywords:** authentication, cheese, elemental metabolomics, Graviera, PDO, rare earth elements

## Abstract

This study presents the comprehensive elemental profile of Greek Graviera (Gruyère) cheeses. In total, 105 samples from nine different geographic regions produced from sheep, goat and cow milk and their mixtures were assessed. Elemental signatures of 61 elements were investigated for determination of geographic origin and milk type. Regional and milk type classification through Linear Discriminant Analysis was successful for almost all cases, while a less optimistic cross validation exercise presented lower classification rates. That points to further research using a much larger sample set, increasing confidence for cheese authentication utilizing also bioinformatics tools under development. This is the first study reporting signatures of 61 elements in dairy products including all sixteen rare earth elements and all seven precious metals. Safety and quality were assessed regarding toxic and nutritive elements. According to both EU and USA regulations and directives, Graviera is a nutritional source for trace and macro elements with low levels of toxic elements.

## 1. Introduction

Food authentication’s importance is increasing due to the consumers’ interest in accurate food labeling, forcing producers and retailers to follow. Regulatory authorities are interested in analytical methods for food authenticity to support law enforcement [1,2]. Dairy products play a central role in a nutritious and balanced diet and their consumption has been associated with several health benefits due to their high contents of protein, essential fatty acids and minerals [3]. 

“Graviera” (*Gruyère*) is a hard type cheese, holding the second place, after “Feta”, in the Greek dairy production volume. It is produced mainly from a mixture of sheep and goat milk although Gravieras can be found made of solely sheep, or goat or cow or a mixture of all three kinds of milk. Most of the 700 Greek cheese-making dairies are small-medium size, collecting milk over a radius of about 30 km, although very few can collect milk over substantially longer distances. The majority of dairy sheep and goat farms are also of small to medium size, grazing their animals near the farm [4]. Most of the Gravieras are commercialized with a geographical denomination, but only three of them are registered under the Protected Designation of Origin (PDO) EU scheme, namely: “Graviera Agrafon”, “Graviera Kritis” and “Graviera Naxou”. However, the variety of local climates, the highly rich flora of endemic plants, the predominant microflora, and the processing of the milk along with the traditional cheese-making practices are crucial for Graviera’s quality characteristics [5]. 

Authenticity of dairy products can be assessed by various analytical techniques such as molecular [6], chromatographic [7], vibrational and fluorescence spectroscopy [8], elemental fingerprinting [9,10,11], isotopic [12], non-chromatographic mass spectrometry [13] and Nuclear Magnetic Resonance (NMR) [14]. Cheese authentication through elemental fingerprinting has been highlighted in several articles [9,10,11,15,16,17,18]. However, these publications do not take into account the comprehensive elemental profile as proposed by elemental metabolomics, including Rare Earth Elements (REE) and precious metals [19].

Results from earlier studies reveal a relation between the elemental profile of soil and the derived food products [15,20]. The elemental content of animal products depends, beyond feed-vegetation intake, on various factors such as animal species (e.g., cow, sheep or goat), mineral supplementation, drinking water and production practices. Moreover, the elemental profile of soil and mineral pollution are associated with each specific geographical area and characterize the origin [9,12,21,22]. 

Elemental transfer along the food chain is a complex procedure that is also element specific. Elemental fingerprint in the first part of the food chain, i.e., the “plant”, might be substantially different from the elemental fingerprint in the last part, i.e., milk, meat or cheese. That is due to the impact of the aforementioned factors, all metabolic processes and different absorption rates in different organisms. In this respect, the REE fingerprint is directly linked to the geology of the area and could be minimally affected by other factors. REEs have been proven to be very reliable and authentic markers in various products, like split peas [23], wines [24] and game meat [25] and are minimally affected by harvesting variations [26]. 

Elemental metabolomics is emerging as a new technology with applications in various fields such as nutrition, agriculture and links food science to health [19]. Inductively coupled plasma mass spectroscopy (ICP-MS) is the main choice due to its capabilities for rapid ultra-trace level multi-element determinations. Basic principles of elemental metabolomics include but are not limited to proper sample preparation for elemental analysis, use of standard reference materials during ICP-MS analysis and proper data handling, statistical analysis and reporting. Elemental metabolomics Standard Operation Principles (SOPs), as well as the minimal information reporting standards (MIRSs), are reported in literature [19].

This study determined, through ICP-MS analysis, the elemental signatures of 61 elements of Greek Graviera cheese with the aim to investigate the use of the elemental metabolome for the assessment of geographical origin, safety and nutritional quality of this type of cheese.

## 2. Results & Discussion

### 2.1. Elemental Concentrations in Greek Graviera and Implications for Authentication

#### 2.1.1. Geographical Origin

The database of elemental signatures, comprised of 61 elements, is shown Table 1, Table 2, Table 3 and Table 4. Table 1 presents rare earth elements & actinides (16 & 2 = 18 elements), Table 2 precious metals & ultra-trace elements (7 & 8 = 15 elements), Table 3 trace elements (15 elements) and Table 4 trace elements of high abundance & macro elements (9 & 4 = 13 elements). Ten rare earth elements (Dy, Er, Eu, Nd, Pr, Sc, Sm, Y, Yb) and one actinide (U) showed statistically significant different values between Greek regions (administrative regions). The same was found for 4 precious metals (Au, Pd, Re, Ru) and 5 ultra-trace elements (Nb, Ta, Tl, W, Zr). All 28 trace and macro elements showed statistically significantly different values between Greek regions in addition to Ag, Al, Bi, Cd, Cu, Mo, Ni and Pb. 

Sixty-one elements were used as predictor variables to develop a method for assigning Graviera origin to nine geographic regions. The classification table (Table 5) shows that one sample from the Epirus region (sample 15, Table S1, from Arta) and another from Thessaly (sample 98, Table S1, from Larisa) were misclassified as coming from Central Greece. However, both Arta and Larisa are adjacent to the Central Greece region. That could provide an adequate explanation for the misclassification as goats and sheep movement between adjacent regions could modify their elemental content and subsequently the milk they produce. However, after further investigation we found that the two dairies in Arta and Larisa, to fulfill their needs, purchased milk from Central Greece, Amfiloxia and Lamia, respectively. The third misclassified sample was from Macedonia region (sample 45, Table S1, from Grevena, prepared from cow milk) classified as South Aegean. Cows in the Macedonia region obtain a large portion of their feed from grass that grows locally that is rich in REEs and aluminum [27] (aluminum ores are usually accompanied by REEs [28]). This is reflected in the content of REEs and aluminum found in Gravieras produced in that region. Sample 45 shows less than 50% REEs and aluminum content compared to all other samples from this region. This points to a different feeding scheme using mostly imported feeds as in the South Aegean. Through a cross validation exercise using the leave-one-out approach, the above results are quite optimistic and the classification rate was just 32.7%. This warrants further research enhancing the sample bank with much more samples resulting from different production periods. 

Elemental metabolomics has potential for detecting production method (feed with pasture vs imported/dried feeds). This needs further research with feeding experiments. A useful aspect of elemental metabolomics applied to dairy products could be a bioinformatics tool to detect the feeding scheme utilizing soil composition analysis. Details on the tool/algorithm can be extracted from the discussion above on the Macedonia region’s cows and soil.

The most significant predictor variables are the rare earths Ce, Er, Eu, Ho, La, Sm, Tm, Yb, the actinide Th, the precious metals Pt, Re, Ru, the ultra trace elements Hf, Nb, Sb, W, the trace elements Ag, Al, As, B, Ba, Cd, Co, Cr, Cs, Fe, Ga, Hf, Mo, Nb, Ni, Pb, Sb, Se, Sr, V, W, Zn and the macro elements Ca, Mg and P The classification was more successful using the comprehensive elemental signature as proposed by elemental metabolomics [19]. This result on cheeses is in contrast to previous studies on authentication of game meat [25], wines [24] and split-peas [23], where specific groups of elements such as REEs were sufficient.

Levels of REEs were higher in Crete cheeses, probably reflecting the vegetation and soil composition [21]. This is most pronounced for the light REEs (LREEs) Pr and Nd (Table 1). This is in accordance with previous findings that Crete is enriched in LREEs, due to monazite and allanite ores [29]. It is interesting to note that another couple of LREEs, Eu & Sm, were enriched, by three times, in cheese from Central Greece, pointing to further authentication markers. These findings about Eu and Sm need further research such as a check of soil composition differences and the influence of different flora grown there.

Usually all rare earths in different materials follow the same pattern, i.e., they are all enriched or depleted: fava Santorini’s [26]; Italian milk [30]; mushrooms substrates [31]. However, this pattern is differentiated by genetic factors as seen in two different mushroom species [31]. The production method could also differentiate the pattern as seen in game and farmedrabbits [25].

North Aegean cheeses showed much higher levels of Rb, Cs (alkali metals) and Sr, Ba (alkaline earth metals) in agreement with previous studies [9,10,11,12,15,18,32,33,34], where alkali and alkaline earth metals, especially Rb, Cs, Sr and Ba, were proven reliable cheese authenticity markers. Another interesting result shown in Table 2 is that the precious metals Au, Pd & Ru were found in higher amounts in Thessaly’s cheeses. Geological data [35] explain the increased content of precious metals transported from the Pindus mountain range by the Pinios River to Thessaly. This is in accordance with the view that precious metals are potential authenticity markers [22]. 

Our data are in line with previous findings. Camin et al. also stated [12] that levels of Cu, Mo, Ni, Fe, Mn, Ga and Se showed significant differences between grated hard cheeses. Osorio et al. [15] found that Ag, Ba, Ca, K, Mg, Mn, P and Sr presented different profiles for different Halloumi cheese production locations and also highlighted the potential of Sr for traceability information from soil as it cannot be added from cheese making equipment. All elements commended by Osorio in addition to Ag presented significant differences between regions in our work. Korenovska and Suhaj [11], working with Slovakian, Polish, and Romanian Bryndza cheeses, found also that Cr, Hg, Mn and V along alkali and alkaline earth metals were the best elemental indicators.

In agreement with our results, Pillonel et al. [18] working with Emmental cheese support the view that the elemental profile allows the discrimination of close regions of production, where the distances are in the order of a few tenths of kilometers up to 150 km. It should be noted that the distance range for adjacent regions in our study is between 10 to 150 km. This is the first study reporting precious metals, rare earth and ultratrace element assessment for dairy product authentication.

#### 2.1.2. Milk Type

The Gravieras used in this study were manufactured from mixed sheep and goat milk (78), sheep (10), goat (8) and cow (8) and one from sheep, goat and cow milk. The comprehensive elemental fingerprint (Table S2) of 61 elements was used for the classification according to the milk type. Best markers of milk type were: Bi, Cr, Fe, Mn, Ni, Se, Sr, Zn, Mg and P (Table S2). The classification table (Table 6), shows that all 78 cheeses from sheep and goat milk were correctly categorized. Our data did not contain information on the % percentage of goat milk used. This explains the misclassification of two out of the ten sheep milk Graviera samples into the sheep and goat class. This group is not so well defined and homogenous as even the same producer uses different percentages of goat milk, according to its availability. Only one cow Graviera sample was misclassified into the sheep + goat group. This Graviera was from the Macedonia region (sample 41, Table S1, from Grevena). Through a cross validation exercise using the leave-one-out approach, the above results are quite optimistic and the classification rate was only 50.5%.

Previous attempts to classify cheeses according to the milk type, in comparison to our study, were restricted to small elemental fingerprints. Fresno et al. [34] were the first to report differences in P, K, Mg, Zn, Fe and Mn, concerning various ripened and unripe Spanish cheeses. Necemer et al. [32] found that, for Slovenian cheeses, the best milk-type indicators were Ca, Br, Zn and Sr. Our study is the first concerning the determination of cheese milk type using elemental metabolomics.

### 2.2. Contribution to Total Diet—Safety Aspects and Nutritional Value

Regarding toxic elements such as, Cd, Pb, Sn and Sb, the examined samples presented low values for most of them. In more detail, Pb levels were determined to range from 19.8 μg kg^−1^ (North Aegean) to 38.3 μg kg^−1^ (Central Greece), i.e., similar to the Khozam et al. study [36] on Lebanese cheese (32.4 μg kg^−1^ wet weight), but noticeably lower than that reported by Vural et al. study [37] for south-eastern Anatolia-Turkey cheese (4600–7700 μg kg^−1^ wet weight), the Lante et al. study [38] for Crescenza and Squacquerone cheeses (600 μg kg^−1^ fresh weight) and the Mendil et al. study [39] for Turkish cheeses (110–960 μg kg^−1^ wet weight). 

Concerning As levels, they were determined to range from 171 μg kg^−1^ (Thessaly) to 247 μg kg^−1^ (North Aegean). Khozam et al. [36] found lower values in Lebanese cheese (2.2 μg kg^−1^ wet weight). Regarding Cd concentration, it ranged from 3.8 μg kg^−1^ (Thrace) to 6.2 μg kg^−1^ (Kriti) in the present study, i.e., lower levels compared to Vural et al. [37] (100 μg kg^−1^ to 300 μg kg^−1^ wet weight). Further, Khozam et al. [36] determined Cd in Lebanese cheese to be 0.14 μg kg^−1^ (wet weight). As regards Sn levels, in the present study they were determined to range from 7.0 μg kg^−1^ (Makedonia) to 13.4 μg kg^−1^ (Peloponnisos), while in the Khozam et al. study [36], they were much lower, i.e., 0.037 μg kg^−1^ (wet weight). Finally, concerning Sb levels, they were measured to range from 4.4 μg kg^−1^ (Makedonia) to 6.5 μg kg^−1^ (North Aegean), whereas Khozam et al. [36] determined them to be 0.44 μg kg^−1^ (wet weight). Regarding other elements, Greek Graviera samples presented lower levels of Thallium, Vanadium, Silver, Antimony, Aluminium and higher levels of Titanium and Barium in comparison to Turkish milk and yogurt [40]. As mentioned above, Hg was not determined. 

The Food and Agriculture Organization/World Health Organization Joint Expert Committee on Food Additives (JECFA) has established a Provisional Tolerable Weekly Intake (PTWI) for several toxic elements and especially heavy metals. According to the Hellenic Statistical Authority, the daily intake of cheese in the Greek population is 94.7 g/person. For an adult person (e.g., a man of 75 kg body weight), the percentage intake of each toxic element is provided in Table 7. The highest intake is observed for As (12.8%) while the lowest is for Sn (0.0007%), reflecting the high allowed limit for Sn. Column 4 shows the % intake according to the Reasonable Daily Intake of cheese based on the Canadian Food Inspection Agency, 57 g cheese consumption per day. Here, it must be mentioned that reasonable intake has been estimated considering the food habits of Canadians. As regards for these calculations, a mean value of all analyzed Graviera cheese samples was taken into account for each element.

Concerning nutrition, trace amounts of Fe, Mn, Mo, Zn, Co, Ni, Cr, Se, Cu, Si, I, and F are necessary for proper human health, apart from H, C, N, O, Na, K, S, Cl, Mg, Ca, and P which are required in relatively large quantities in a diet. There is also a group of elements called ultra-trace minerals, including V, Sn, Ni, As, and B, that are being investigated for possible biological function but currently do not have clearly defined biochemical roles [19]. Thus, in order to prevent nutrient deficiencies, but also to reduce the risk of chronic diseases such as osteoporosis, cancer and cardiovascular disease, scientific food committees around the world have established specific limits for each element intake with values adapted to different population groups (children, adolescents, pregnant women or older people). The European Commission has established Nutritive Reference Values for adults, according to Regulation (EU) No 1169/2011 (25 October 2011) that are presented in Table 8. WHO/FAO and USDA (United States Department of Agriculture) have also established Recommended Dietary Allowances (RDA) indicating the amount of an individual nutrient that people need for good health depending on their age and gender. 

As shown in Table 8, % Ca intake from Graviera was sufficient and ranged from 54% to 111%, while P ranged from 56% to 94%. Moreover, the Ca–P ratio was 1.4:1, so consumption of Graviera cheese is one the most convenient ways for proper intake of both minerals through the diet. High dietary Ca–P ratios play important role in bone health [41]. The % zinc intake ranged from 16% to 31%. Iron, Cr and Mo % intakes ranged from 6.2% to 35%, 74% to 185% and 12% to 22%, respectively. Regarding Mg, Cu, Mn and Se the % intake is less significant. These results highlight Graviera cheese as good source of trace and macro elements, especially for Ca, P, Zn, Cr, Fe and Mo.

Compared with other studies like the Moreno-Rojas et al. study of different cheese types [10], nutritive elements were found at similar levels. Camin et al. [12] found lower Fe and higher Se and Mo in various European hard cheeses such as PDO Parmigiano Reggiano. Suhaj et al. [33] determined Cr in lower levels, Mo and Ca in slightly lower levels and Mn in slightly higher levels in some European Emmental and Edam hard cheeses than the Graviera samples in our study. 

## 3. Materials and Methods

### 3.1. Instrumentation and Reagents

Chemicals used were nitric acid (Suprapur^®^, 65% *w/v*, Merck, Darmstadt, Germany), hydrogen peroxide (Suprapur^®^, 30% *w/v*, Merck, Darmstadt, Germany), ICP internal standards of Ge and In and ICP-MS certified multi-element standards (all from Inorganic Ventures, NJ, USA). Ultrapure water with a resistance of 18.2 MΩ cm^−1^ obtained from a MilliQ plus system (Millipore, Saint Quentin Yvelines, France) was used in all procedures. 

Elemental content was determined using a Perkin Elmer (SCIEX, Toronto, ON, Canada) 9000 Series ICP-MS. Inductively coupled plasma mass spectroscopy is predominantly used in authentication studies due to its capability for rapid ultra-trace level multi-element determinations [42].

### 3.2. Sample Collection, Preparation and Digestion

**Figure 1:**
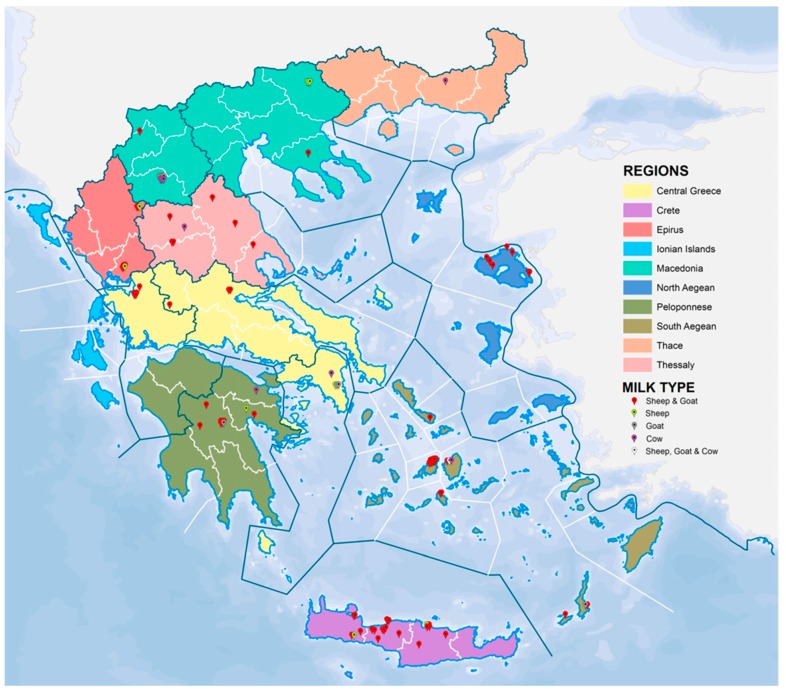
Map depicting origin of samples.

One hundred and five Graviera cheese samples were used for the purposes of this study. The geographical origin of the samples is reported in Table S1 and depicted in the map shown in Figure 1. Most of the samples were collected from small-medium dairies and the rest from the respective local markets. The sampling strategy excluded large dairies that are able to collect milk from different Greek regions and bulk it in their premises for the production of their own trade mark. Samples were taken from a lot and after grinding, they were preserved in a freezer (−32 °C) before analysis. 

Sample digestion was performed with a microwave-assisted digestion system (CEM, Mars X-Press, Matthews, NC, USA). Approximately 0.50 g of cheese was weighted in an analytical balance in a polypropylene tube. Then, 4.0 mL of HNO_3_ was added to pre-digest samples for 30 min. The resulting cheese suspension was transferred quantitatively, with the use of 4.0 mL HNO_3_ and 2.0 mL H_2_O_2_ to the microwave digestion PTFE vessel. The samples were heated in the microwave accelerated digestion system according the following program: the power was ramped during 20 min from 100 to 1200 W and held for 15 min. The temperature reached a maximum of 200 °C and followed by a cool-down cycle for 15 min. PTFE vessels were sealed throughout the aforementioned cycle to avoid volatilization losses. Although all samples were completely brought to solution, to disregard any small particle passing optical inspection entering the ICP-MS, solutions were filtered with polyester disposable syringe filters 0.20 μm/ 15 mm (Chromafil, Macherey-Nagel, Düren, Germany). Before injection in the ICP-MS, sample solutions were diluted, as required, with ultrapure water. 

### 3.3. ICP-MS Analysis

The studied elements assessed were:

- REEs: Ce, Dy, Er, Eu, Gd, Ho, La, Lu, Nd, Pr, Sc, Sm, Tb, Tm, Y, Yb
- Actinides: Th, U
- Precious metals: Au, Ir, Pd, Pt, Re, Rh, Ru
- Ultra-trace elements: Hf, Nb, Sb, Sn, Ta, Tl, W, Zr
- Trace elements: Ag, Al, As, B, Ba, Bi, Cd, Co, Cr, Cs, Cu, Fe, Ga, Mn, Mo, Ni, Pb, Rb, Se, Si, Sr, Ti, V, Zn
- Macro elements: Ca, K, Mg, P

Limits of quantification for all were lower than those determined in the samples (Table S3). Operating conditions of the ICP-MS were as follows: nebulizer gas flow of 0.75 L min^−1^, ICP RF power of 950 W, lens voltage of 7 V, pulse stage voltage of 950 V and sample uptake rate of 26 rpm. Calibration curves ranges were from 1 ng kg^−1^ to 1000 μg kg^−1^ for rare earths, precious metals and ultra-trace elements, while from 0.01 μg kg^−1^ to 10 mg kg^−1^ for trace and macro elements. Indium was used as internal standard for rare earths, precious and ultra-trace elements, while germanium was used for trace and macro elements. In detail, the daily analytical procedure is:

➢ Start
  - Daily performance check with set up solution from Perkin Elmer that contains 10 μg L^−1^ of the following elements: Be, Mg, Co, In, Pb
  - Standard solutions for REEs, actinides, precious metals and ultra-trace elements of 0.001, 0.01, 0.1, 0.5, 1, 5, 10, 50, 100 and 1000 μg L^−1^
  - Standard solutions for macro and trace elements of 0.01, 0.1, 0.5, 1, 5, 10, 50, 100, 1000 and 10000 μg L^−1^
➢ Steps to be repeated after 4 h:
  - Standard reference materials
  - Blank
  - Samples
➢ End
  - Standard reference materials

### 3.4. Calibration and Quality Assurance

To assess the accuracy of the process the following standard reference materials were obtained from the European Commission, Joint Research Center, institute for reference materials and measurements IRMM, Belgium and the National Institute of Standards & Technology (NIST), USA:

- Trace and macro elements, ERM-BD151 skimmed milk powder (IRMM), RM 8414 bovine muscle powder (NIST) and RM 1573a tomato leaves (NIST).
- Rare earth elements and actinides CRM-668 mussel tissue (IRMM).

The standard reference materials were subjected the same analytical process: Digested three different times, each digestate measured in triplicate (Table 9). Recoveries were in the range 67–121% for all elements other than Se. In order to overcome Ar^2+^ interferences we measured Se 82.

### 3.5. Statistical Analysis

Statistical analysis was performed using SPSS software (IBM, Armonk, NY, USA) for the descriptive statistics and cross validation and Statgraphics Centurion XV software (Statpoint technologies, Warrenton, VA, USA) in order to analyze the data using statistical models and predictive analyses (Linear Discriminant Analysis). 

## 4. Conclusions

We present results from 61 elements in cheese for the first time with implications in food authentication, safety and nutrition. Further work is in progress for data selection, increasing confidence for food authentication using bioinformatics tools under development. This is the first study reporting signatures of 61 elements including rare earth elements and all the precious metals in cheese. We highlight the application of elemental metabolomics to human nutrition assessing both nutritive and toxic elements. The results demonstrate that elemental metabolomics could be potentially used for discrimination of cheeses produced in different geographical zones and milk type. The method needs further improvement by bioinformatics tools to automate data cleaning done manually.

In comparison to molecular analysis, elemental metabolomics is simple and convenient. The first step is accurately weighing samples in capped polypropylene tubes to analyze when convenient, when adequate samples are collected and when instrumentation is available. There are no requirements concerning temperature, time, or any other storage condition. The only requirement is creation of comprehensive elemental metabolome databases for food authentication, quality and safety. Elemental metabolomics are becoming more affordable by lowering the ICP-MS purchasing cost and increasing capabilities concerning interferences [19]. We envisage open access elemental databases for improvement of human nutrition and health. 

## Figures and Tables

**Table 1:** Rare earth elements and actinides in Greek Graviera, μg kg^−1,^ mean ± Standard Error of the Mean, SEM (samples).

Region		Ce	Dy	Er	Eu	Gd	Ho	La	Lu	Nd	Pr	Sc	Sm	Tb	Tm	Y	Yb	Th	U
Central Greece	Mean	2.5	0.17	0.19	3.1	0.5	0.06	1.7	0.07	1.2	0.33	26	2.9	0.16	0.07	0.8	0.22	1.4	3.7
	SEM (8)	0.5	0.04	0.04	1.0	0.1	0.01	0.4	0.02	0.2	0.07	7	0.7	0.04	0.02	0.2	0.04	0.2	0.4
Crete	Mean	4.1	0.24	0.26	1.0	0.9	0.08	2.7	0.08	2.0	0.5	27	1.0	0.21	0.11	1.1	0.29	1.7	2.6
	SEM (22)	0.9	0.04	0.05	0.1	0.2	0.01	0.6	0.01	0.6	0.1	4	0.1	0.03	0.02	0.4	0.06	0.1	0.3
Epirus	Mean	2.8	0.24	0.31	1.1	0.69	0.086	1.9	0.092	1.6	0.43	34	0.89	0.18	0.097	0.9	0.	1.56	2.2
	SEM (9)	0.3	0.02	0.02	0.09	0.05	0.006	0.2	0.005	0.1	0.04	2	0.06	0.01	0.009	0.1	0.02	0.07	0.1
Macedonia	Mean	2.2	0.18	0.25	0.7	0.56	0.071	1.3	0.085	1.0	0.24	2	0.8	0.25	0.085	0.51	0.25	1.6	2.9
	SEM (13)	0.4	0.02	0.02	0.1	0.06	0.005	0.3	0.006	0.1	0.03	1	0.1	0.04	0.007	0.08	0.02	0.1	0.2
North Aegean	Mean	2.0	0.147	0.217	0.8	0.48	0.065	1.3	0.080	0.9	0.23	26	0.69	0.23	0.078	0.35	0.21	1.60	2.7
	SEM (8)	0.4	0.007	0.008	0.1	0.05	0.004	0.3	0.003	0.1	0.02	2.0	0.09	0.04	0.005	0.06	0.01	0.07	0.2
Peloponnese	Mean	3.6	0.26	0.28	1.2	0.7	0.09	1.8	0.09	1.5	0.45	28	1.3	0.18	0.08	1.2	0.28	1.7	3.3
	SEM (11)	0.7	0.04	0.04	0.2	0.1	0.01	0.3	0.01	0.2	0.08	5	0.1	0.03	0.01	0.2	0.03	0.2	0.4
South Aegean	Mean	3.7	0.22	0.26	1.1	1.0	0.09	2.3	0.086	1.7	0.41	28	1.1	0.19	0.09	0.9	0.28	1.52	2.4
	SEM (21)	0.8	0.02	0.04	0.1	0.3	0.01	0.4	0.007	0.3	0.07	3	0.2	0.02	0.01	0.2	0.03	0.09	0.3
Thessaly	Mean	2.2	0.12	0.13	0.9	0.7	0.06	3	0.07	0.7	0.18	19	1.4	0.12	0.05	0.58	0.16	1.3	4.2
	SEM (7)	0.4	0.02	0.02	0.2	0.3	0.01	2	0.01	0.1	0.02	5	0.3	0.03	0.02	0.08	0.03	0.2	0.6
Thrace	One sample **	1.7	0.13	0.07	0.6	0.3	0.03	0.9	0.04	0.5	0.16	14	1.0	0.06	0.02	0.8	0.12	1.2	4.3
*p* Value *		0.098	>0.001	0.001	>0.001	0.190	0.075	0.209	0.413	0.002	>0.001	0.028	>0.001	0.236	0.075	0.004	0.007	0.337	>0.001

* *p* Values > 0.05 mean that the element concentration is not statistically different between regions. ** One digestion, measured in triplicate.

**Table 2:** Precious metals and ultra-trace elements in Greek Graviera, μg kg^−1^, mean ± Standard Error of the Mean, SEM (samples).

Region		Au	Ir	Pd	Pt	Re	Rh	Ru	Hf	Nb	Sb	Sn	Ta	Tl	W	Zr
Central Greece	Mean	6	0.9	2.1	1.4	1.6	5	10	0.5	1.2	6	12	0.9	2.0	6	7
	SEM (8)	1	0.1	0.4	0.3	0.4	2	2	0.1	0.3	1	4	0.3	0.6	2	1
Crete	Mean	4.0	0.65	1.7	1.6	0.7	5.6	6	0.52	1.2	6	10	0.7	1.32	7	5
	SEM (22)	0.8	0.07	0.3	0.2	0.2	0.8	1	0.06	0.2	1	2	0.2	0.08	1	1
Epirus	Mean	3.8	0.68	1.7	1.73	2.2	4.3	5.3	0.56	1.30	6.2	13	0.50	1.5	7.6	5.4
	SEM (9)	0.4	0.04	0.1	0.07	0.6	0.4	0.2	0.04	0.09	0.4	2	0.03	0.1	0.5	0.4
Macedonia	Mean	3.8	0.64	1.34	1.6	0.6	5	3.8	0.49	0.6	4.4	7	0.44	1.4	4.4	3.6
	SEM (13)	0.5	0.04	0.09	0.1	0.2	2	0.7	0.03	0.1	0.2	1	0.08	0.1	0.6	0.3
North Aegean	Mean	3.1	0.57	1.37	1.5	0.5	6	4.0	0.47	0.89	5.0	9	0.50	1.31	5.4	3.8
	SEM (8)	0.3	0.04	0.09	0.1	0.1	1	0.5	0.02	0.09	0.2	2	0.07	0.05	0.5	0.3
Peloponnese	Mean	6	0.9	2.9	1.5	0.8	4.7	7.1	0.6	1.3	6.2	13	0.6	1.5	6.0	6.0
	SEM (11)	2	0.1	0.2	0.2	0.2	0.9	0.8	0.1	0.2	0.7	4	0.1	0.1	1	0.5
South Aegean	Mean	3.7	0.64	1.8	1.8	0.8	5.1	5.8	0.57	1.3	6.1	11	0.51	2.1	6.5	5.6
	SEM (21)	0.6	0.04	0.2	0.1	0.1	0.7	0.9	0.05	0.1	0.6	2	0.07	0.3	0.9	0.8
Thessaly	Mean	7	0.7	2.2	1.4	1.2	4	11	1.1	1.8	5.8	13	3	2.1	4	7
	SEM (7)	1	0.1	0.3	0.3	0.4	1	2	0.6	0.7	0.8	3	2	0.2	1	1
Thrace	One sample **	5	0.4	1.4	0.5	0.2	1	11	0.1	0.8	4.6	9	1	1.3	0.9	6
*p* Value *		0.005	0.075	0.015	0.389	0.026	0.845	>0.001	0.111	0.029	0.217	0.419	0.002	0.013	0.022	0.003

* *p* Values > 0.05 mean that the element concentration is not statistically different between regions. ** One digestion, measured in triplicate.

**Table 3:** Trace elements in Greek Graviera, μg kg^−1^, mean ± Standard Error of the Mean, SEM (samples).

Region		Ag	As	Ba	Bi	Cd	Co	Cr	Cs	Cu	Ga	Mo	Ni	Pb	Se	V
Central Greece	Mean	3.4	25	3000	17	5.2	80	650	14	800	35	100	430	38	110	500
	SEM (8)	0.6	20	500	6	0.7	20	40	3	100	4	30	40	17	20	70
Crete	Mean	4.4	212	1000	16	5.8	39	590	4.5	780	13	110	370	28	65	390
	SEM (22)	0.7	8	100	2	0.8	6	30	0.7	50	1	30	20	7	9	20
Epirus	Mean	4.2	202	920	15.3	6.2	36.0	580	5.3	820	12.4	130	380	27	75	339
	SEM (9)	0.3	6	70	0.6	0.3	0.7	10	0.4	40	0.5	10	20	3	3	9
Macedonia	Mean	2.9	220	1000	14.8	4.3	28	500	3.0	530	12	60	290	21	51	470
	SEM (13)	0.2	20	200	0.8	0.2	5	30	0.4	80	2	10	40	4	9	40
North Aegean	Mean	3.8	220	800	14.5	5.4	31	540	2.8	670	11.6	100	320	20	60	440
	SEM (8)	0.4	9	60	0.7	0.3	3	20	0.3	90	0.8	10	30	2	4	30
Peloponnese	Mean	3.9	240	1000	49	5.8	70	640	5	640	17	110	367	24	100	470
	SEM (11)	0.6	10	200	35	0.6	20	30	1	60	2	20	9	4	20	50
South Aegean	Mean	4.0	220	1000	14	5.8	38	570	6	780	14	110	350	32	63	400
	SEM (21)	0.4	10	100	1	0.4	5	20	2	40	2	20	20	6	8	30
Thessaly	Mean	5	170	1155	14	5.3	70	700	5	800	19	80	410	25	110	340
	SEM (7)	2	30	207	2	0.9	10	100	1	100	4	20	30	7	20	60
Thrace	One sample **	2	240	697	11	3.8	85	730	3	740	17	90	420	20	210	560
*p* Value *		0.184	0.044	>0.001	0.416	0.140	>0.001	>0.001	>0.001	0.130	>0.001	0.137	0.058	0.432	>0.001	0.007

* *p* Values > 0.05 mean that the element concentration is not statistically different between regions. ** One digestion, measured in triplicate.

**Table 4:** Trace elements of high abudance mg kg^-1^ and macro elements (Ca, K, Mg & P) g kg^-1^, mean ± Standard Error of the Mean, SEM (samples).

Region		Al	B	Fe	Mn	Rb	Si	Sr	Ti	Zn	Ca	K	Mg	P
Central Greece	Mean	1.6	62	32	0.42	1.5	157	7.6	62	34	8.7	0.56	0.47	7.3
	SEM (8)	0.2	8	3	0.04	0.1	14	0.9	2	2	0.6	0.04	0.02	0.3
Crete	Mean	1.8	48	29	0.39	0.98	134	4.7	62	30	9.9	0.45	0.52	7.0
	SEM (22)	0.3	5	1	0.02	0.07	8	0.5	1	1	0.4	0.02	0.01	0.2
Epirus	Mean	1.3	42.9	31.9	0.44	1.03	126	3.8	68	34.9	10.9	0.56	0.56	7.5
	SEM (9)	0.2	0.6	0.6	0.02	0.06	3	0.3	1	0.8	0.2	0.02	0.01	0.1
Macedonia	Mean	1.3	44	23	0.32	0.8	128	2.4	50	28	7.4	0.7	0.38	5.8
	SEM (13)	0.2	4	2	0.03	0.1	10	0.3	5	4	0.8	0.1	0.03	0.5
North Aegean	Mean	0.9	44	26	0.27	0.63	122	2.5	56	35	8.7	0.68	0.39	6.3
	SEM (8)	0.1	2	1	0.02	0.04	4	0.2	2	1	0.5	0.03	0.02	0.3
Peloponnese	Mean	2	58	29	0.47	0.90	151	3.6	62	33	9.3	0.64	0.50	7.2
	SEM (11)	1	6	1	0.05	0.08	11	0.3	1	1	0.6	0.06	0.02	0.2
South Aegean	Mean	1.8	45	31	0.36	0.88	124	4.2	61	32	10.0	0.51	0.49	6.9
	SEM (21)	0.4	3	1	0.03	0.06	5	0.4	2	1	0.4	0.02	0.03	0.2
Thessaly	Mean	1.6	79	35	0.41	1.02	166	4.3	68	36	9.8	0.66	0.55	8.1
	SEM (7)	0.3	19	3	0.04	0.08	30	0.5	2	1	0.8	0.06	0.03	0.4
Thrace	One sample **	3.0	85	40	0.29	0.65	177	3.7	66	42	7.4	0.70	0.41	8.3
*p* Value *		0.162	>0.001	>0.001	>0.001	>0.001	0.009	>0.001	>0.001	0.016	>0.001	0.020	>0.001	>0.001

* *p* Values > 0.05 mean that the element concentration is not statistically different between regions. ** One digestion, measured in triplicate.

**Table 5:** Geographical region classification table based on the 61 element signature.

Actual	Group Size	Predicted								
		Central Greece	Crete	Epirus	Macedonia	North Aegean	Peloponnese	South Aegean	Thessaly	Thrace
Central Greece	13	13	0	0	0	0	0	0	0	0
		(100.0%)	(0.0%)	(0.0%)	(0.0%)	(0.0%)	(0.0%)	(0.0%)	(0.0%)	(0.0%)
Crete	22	0	22	0	0	0	0	0	0	0
		(0.0%)	(100.0%)	(0.0%)	(0.0%)	(0.0%)	(0.0%)	(0.0%)	(0.0%)	(0.0%)
Epirus	9	1	0	8	0	0	0	0	0	0
		(11.1%)	(0.0%)	(88.9%)	(0.0%)	(0.0%)	(0.0%)	(0.0%)	(0.0%)	(0.0%)
Macedonia	13	0	0	0	12	0	0	1	0	0
		(0.0%)	(0.0%)	(0.0%)	(92.3%)	(0.0%)	(0.0%)	(7.7%)	(0.0%)	(0.0%)
North Aegean	8	0	0	0	0	8	0	0	0	0
		(0.0%)	(0.0%)	(0.0%)	(0.0%)	(100.0%)	(0.0%)	(0.0%)	(0.0%)	(0.0%)
Peloponnese	11	0	0	0	0	0	11	0	0	0
		(0.0%)	(0.0%)	(0.0%)	(0.0%)	(0.0%)	(100.0%)	(0.0%)	(0.0%)	(0.0%)
South Aegean	21	0	0	0	0	0	0	21	0	0
		(0.0%)	(0.0%)	(0.0%)	(0.0%)	(0.0%)	(0.0%)	(100.0%)	(0.0%)	(0.0%)
Thessaly	7	1	0	0	0	0	0	0	6	0
		(14.3%)	(0.0%)	(0.0%)	(0.0%)	(0.0%)	(0.0%)	(0.0%)	(85.7%)	(0.0%)

Percent of cases correctly classified: 95.9%.

**Table 6:** Classification table of milk type based on the total elemental fingerprint.

Actual Type of Milk	Group Size	Predicted Type of Milk			
		Sheep + Goat	Sheep	Goat	Cow
Sheep + goat	78	78	0	0	0
		(100.0%)	(0.0%)	(0.0%)	(0.0%)
Sheep	10	2	8	0	0
		(20.0%)	(80.0%)	(0.0%)	(0.0%)
Goat	8	0	0	8	0
		(0.0%)	(0.0%)	(100.0%)	(0.0%)
Cow	8	1	0	0	7
		(12.5%)	(0.0%)	(0.0%)	(87.5%)

Percent of cases correctly classified: 91.9%.

**Table 7:** Provisional Tolerable Weekly Intake (PTWI) of the studied toxic elements along with the % intake in Greek & Canadian populations.

Element	PTWI (μg kg^−1^)	% Intake (Greece)	% Intake (Canada)
Al	1000	1.34%	0.81%
As	15	12.8%	7.7%
Cd	7	0.70%	0.42%
Pb	25	0.91%	0.55%
Sn	14,000	0.0007%	0.0004%

**Table 8:** Nutritive Reference Values (NRV) for adults, Recommended Daily Allowance (RDA) and Adequate Intake (AI) * for male 31–50 years old, female in pregnancy 31–50 years old and lactation 31–50 years old along with % intake according to Greek and Canadian consumptions.

Element	NRV	% Intake		RDA/AI * Males 31–50 years of age	% Intake		RDA/AI * Pregnancy 31–50 years of age	% Intake		RDA & AI * Lactation 31–50 years of age	% Intake	
		Greece	Canada		Greece	Canada		Greece	Canada		Greece	Canada
Ca	800 mg	111	67%	1000 mg	89%	54%	1000 mg	89%	54%	1000 mg	89%	54%
P	700 mg	94%	56%	700 mg	94%	56%	700 mg	94%	56%	700 mg	94%	56%
Mg	375 mg	12%	7.3%	420 mg	11%	6.5%	360 mg	13%	7.6%	320 mg	14%	8.5%
Fe	14 mg	20%	12%	8 mg	35%	21%	27 mg	10%	6.2%	9 mg	31%	19%
Zn	10 mg	31%	19%	11 mg	29%	17%	11 mg	29%	17%	12 mg	26%	16%
Cu	1 mg	6.8%	4.1%	900 μg	7.6%	4.6%	1000 μg	6.8%	4.1%	1300 μg	5.2%	3.2%
Mn	2 mg	1.8%	1.1%	2.3 * mg	1.5%	0.9%	2.0 * mg	1.8%	1.1%	2.6 * mg	1.4%	0.8%
Se	55 μg	13%	7.8%	55 μg	13%	7.8%	60 μg	12%	7.2%	70 μg	10%	6.1%
Cr	40 μg	139%	83%	35 * μg	158%	95%	30 * μg	185%	111%	45 * μg	123%	74%
Mo	50 μg	20%	12%	45 μg	22%	13%	50 μg	20%	12%	50 μg	20%	12%

**Table 9:** Standard reference material results, % recoveries and % RSD, *n* = 9 (three different digestions, measured in triplicate).

	BCR 668					ERM-BD151			
REEs	Certified μg kg^−1^	Found μg kg^−1^	% Recoveries	% RSD	TREs	Certified mg kg^−1^	Found mg kg^−1^	% Recoveries	%RSD
Ce	89 ± 7	82 ± 3	92	4	Cd	0.106 ± 0.013	0.121 ± 0.041	114	34
Dy	8.9 ± 0.6	7.9 ± 0.9	89	11	Cu	5.00 ± 0.23	5.77 ± 0.97	115	17
Er	4.5 ± 0.5	3.7 ± 0.7	82	19	Fe	53 ± 4	50 ± 13	94	26
Eu	2.79 ± 0.16	2.4 ± 0.10	86	4	Mn	0.29 ± 0.03	0.34 ± 0.09	117	26
Gd	13.0 ± 0.6	12.1 ± 0.7	93	6	Pb	0.207 ± 0.014	0.201 ± 0.049	97	24
Ho	1.8 ± 0.6 ^a^	1.2 ± 0.2	67	17	Se	0.19 ± 0.04	0.27 ± 0.09	142	33
La	80 ± 6	71 ± 5	89	7	Zn	44.9 ± 2.3	54.0 ± 7.9	120	15
Lu	0.389 ± 0.024	0.379 ± 0.010	97	3	**Macro elements**				
Nd	54 ± 4	49 ± 1	91	2	Ca	13.9 ± 0.7	9.9 ± 1.4	71	14
Pr	12.3 ± 1.1	11.1 ± 0.2	90	2	K	17.0 ± 0.8	14.0 ± 1.7	82	12
Sc	8.5 ± 1.8	10.3 ± 1.5	121	14	Mg	1.26 ± 0.07	1.20 ± 0.13	95	11
Sm	11.2 ± 0.8	10.4 ± 0.4	93	4	Na	4.19 ± 0.23	3.89 ± 0.7	93	18
Tb	1.62 ± 0.12	1.60 ± 0.07	99	4	P	11.0 ± 0.6	12.3 ± 2.4	112	20
Tm	0.48 ± 0.08	0.571 ± 0.03	119	5					
Y	59 ± 5	53 ± 3	90	6					
Yb	2.8 ± 0.5 ^a^	2.1 ± 0.1	75	5					
**Actinides**									
Th	10.7 ± 1.2	9.7 ± 0.3	91	3					
U	56 ± 5	51 ± 4	91	8					

^a^ Indicative values.

## Supplementary Materials

The following are available online, Table S1: Sample description, Table S2: Mean value and SEM, Standard Error of the Mean (number of samples) of the elements for all milk types. The results are expressed in μg kg^−1^ except for the macro elements, which are expressed in g kg^−1^. Table S3. Mass of quantification, limits of Detection (LoD), limits of Quantification (LoQ) (μg kg^−1^) and coefficient of determination.

## References

[1] Danezis G.P., Tsagkaris A.S., Camin F., Brusic V., Georgiou C.A. (2016). Food authentication: Techniques, trends & emerging approaches. TrAC Trends Anal. Chem..

[2] Danezis G.P., Tsagkaris A.S., Brusic V., Georgiou C.A. (2016). Food authentication: State of the art and prospects. Curr. Opin. Food Sci..

[3] Karoui R., Toldrá F. (2017). Methodologies for the Characterization of the Quality of Dairy Products. Advances in Food and Nutrition Research.

[4] Hadjigeorgiou I. (2011). Past, present and future of pastoralism in Greece. Pastoralism.

[5] Litopoulou-Tzanetaki E., Tzanetakis N. (2014). The Microfloras of Traditional Greek Cheeses. Microbiol Spectr.

[6] Cunha J.T., Ribeiro T.I.B., Rocha J.B., Nunes J., Teixeira J.A., Domingues L. (2016). RAPD and SCAR markers as potential tools for detection of milk origin in dairy products: Adulterant sheep breeds in Serra da Estrela cheese production. Food Chem..

[7] Fontenele M.A., Bastos M.D.S.R., dos Santos K.M.O., Bemquerer M.P., do Egito A.S. (2017). Peptide profile of Coalho cheese: A contribution for Protected Designation of Origin (PDO). Food Chem..

[8] Brandao M.P., de Carvalho dos Anjos V., Bell M.J.V. (2017). Time resolved fluorescence of cow and goat milk powder. Spectrochim Acta A Mol. Biomol. Spectrosc..

[9] Moreno-Rojas R., Cámara-Martos F., Sánchez-Segarra P.J., Amaro-López M.A. (2012). Influence of manufacturing conditions and discrimination of Northern Spanish cheeses using multi-element analysis. Int. J. Dairy Technol..

[10] Moreno-Rojas R., Sánchez-Segarra P.J., Cámara-Martos F., Amaro-López M.A. (2010). Multivariate analysis techniques as tools for categorization of Southern Spanish cheeses: Nutritional composition and mineral content. Eur. Food Res. Technol..

[11] Koreňovská M., Suhaj M. (2007). Identification of Slovakian, Polish, and Romanian bryndza cheeses origin by factor analysis of some elemental data. Eur. Food Res. Technol..

[12] Camin F., Wehrens R., Bertoldi D., Bontempo L., Ziller L., Perini M., Nicolini G., Nocetti M., Larcher R. (2012). H, C, N and S stable isotopes and mineral profiles to objectively guarantee the authenticity of grated hard cheeses. Anal. Chim. Acta.

[13] Russo R., Rega C., Chambery A. (2016). Rapid detection of water buffalo ricotta adulteration or contamination by matrix-assisted laser desorption/ionisation time-of-flight mass spectrometry. Rapid Commun. Mass Spectrom..

[14] Mazzei P., Piccolo A. (2012). 1H HRMAS-NMR metabolomic to assess quality and traceability of mozzarella cheese from Campania buffalo milk. Food Chem..

[15] Osorio M.T., Koidis A., Papademas P. (2015). Major and trace elements in milk and Halloumi cheese as markers for authentication of goat feeding regimes and geographical origin. Int. J. Dairy Technol..

[16] Brescia M.A., Monfreda M., Buccolieri A., Carrino C. (2005). Characterisation of the geographical origin of buffalo milk and mozzarella cheese by means of analytical and spectroscopic determinations. Food Chem..

[17] Karoui R., Dufour É., Pillonel L., Picque D., Cattenoz T., Bosset J.O. (2004). Determining the geographic origin of Emmental cheeses produced during winter and summer using a technique based on the concatenation of MIR and fluorescence spectroscopic data. Eur. Food Res. Technol..

[18] Pillonel L., Badertscher R., Froidevaux P., Haberhauer G., Hölzl S., Horn P., Jakob A., Pfammatter E., Piantini U., Rossmann A. (2003). Stable isotope ratios, major, trace and radioactive elements in emmental cheeses of different origins. LWT-Food Sci. Technol..

[19] Zhang P., Georgiou C.A., Brusic V. (2017). Elemental metabolomics. Briefings Bioinf..

[20] Franke B.M., Haldimann M., Gremaud G., Bosset J.-O., Hadorn R., Kreuzer M. (2008). Element signature analysis: Its validation as a tool for geographic authentication of the origin of dried beef and poultry meat. Eur. Food Res. Technol..

[21] Baroni M.V., Podio N.S., Badini R.G., Inga M., Ostera H.A., Cagnoni M., Gallegos E., Gautier E., Peral-García P., Hoogewerff J. (2011). How much do soil and water contribute to the composition of meat? A case study: Meat from three areas of Argentina. J. Agric. Food Chem..

[22] Danezis G.P., Papachristidis C.A., Georgiou C.A., Georgiou C.A., Danezis G.P. (2017). Elemental Fingerprinting. Food Authentication: Management, Analysis and Regulation.

[23] Drivelos S.A., Higgins K., Kalivas J.H., Haroutounian S.A., Georgiou C.A. (2014). Data fusion for food authentication. Combining rare earth elements and trace metals to discriminate “fava Santorinis” from other yellow split peas using chemometric tools. Food Chem..

[24] Pii Y., Zamboni A., Dal Santo S., Pezzotti M., Varanini Z., Pandolfini T. (2017). Prospect on Ionomic Signatures for the Classification of Grapevine Berries According to Their Geographical Origin. Front. Recent Dev. Plant Sci..

[25] Danezis G.P., Pappas A.C., Zoidis E., Papadomichelakis G., Hadjigeorgiou I., Zhang P., Brusic V., Georgiou C.A. (2017). Game meat authentication through rare earth elements fingerprinting. Anal. Chim. Acta.

[26] Drivelos S.A., Danezis G.P., Haroutounian S.A., Georgiou C.A. (2016). Rare earth elements minimal harvest year variation facilitates robust geographical origin discrimination: The case of PDO “Fava Santorinis”. Food Chem..

[27] Melfos V., Voudouris P.C. (2012). Geological, mineralogical and geochemical aspects for critical and rare metals in Greece. Minerals.

[28] Chang C., Li F., Liu C., Gao J., Tong H., Chen M. (2016). Fractionation characteristics of rare earth elements (REEs) linked with secondary Fe, Mn, and Al minerals in soils. Acta Geochim..

[29] Koepke J., Seidel E. (2004). Hornblendites within ophiolites of Crete, Greece: Evidence for amphibole-rich cumulates derived from an iron-rich tholeiitic melt. Ofioliti.

[30] Aceto M., Musso D., Calà E., Arieri F., Oddone M. (2017). Role of Lanthanides in the Traceability of the Milk Production Chain. J. Agric. Food Chem..

[31] Koutrotsios G., Danezis G.P., Georgiou C.A., Zervakis G.I. (2018). Rare earth elements concentration in mushroom cultivation substrates affects the production process and fruit-bodies content of Pleurotus ostreatus and Cyclocybe cylindracea. J. Sci. Food Agric..

[32] Nečemer M., Potočnik D., Ogrinc N. (2016). Discrimination between Slovenian cow, goat and sheep milk and cheese according to geographical origin using a combination of elemental content and stable isotope data. J. Food Compos. Anal..

[33] Suhaj M., Koreňovská M. (2008). Study of some European cheeses geographical traceability by pattern recognition analysis of multielemental data. Eur. Food Res. Technol..

[34] Fresno J.M., Prieto B., Urdiales R., Sarmiento R.M., Carballo J. (1995). Mineral content of some Spanish cheese varieties. Differentiation by source of milk and by variety from their content of main and trace elements. J. Sci. Food Agric..

[35] Economou-Eliopoulos M., Tsoupas G., Kiousis G. (2013). Exploration for platinum-group elements (PGE) in various geotectonic settings of Greece. J. Virtual Explorer.

[36] Khozam R.B., Pohl P., Ayoubi B.A., Jaber F., Lobinski R. (2012). Toxic and essential elements in Lebanese cheese. Food Addit. Contam. Part B Surveill..

[37] Vural A., Narin I., Erkan M.E., Soylak M. (2008). Trace metal levels and some chemical parameters in herby cheese collected from south eastern Anatolia-Turkey. Environ. Monit. Assess..

[38] Lante A., Lomolino G., Cagnin M., Spettoli P. (2006). Content and characterisation of minerals in milk and in Crescenza and Squacquerone Italian fresh cheeses by ICP-OES. Food Control.

[39] Mendil D. (2006). Mineral and trace metal levels in some cheese collected from Turkey. Food Chem..

[40] Şanal H., Güler Z., Park Y.W. (2011). Profiles of non-essential trace elements in ewe and goat milk and their yoghurt, torba yoghurt and whey. Food Addit. Contam. Part. B.

[41] Lee A.W., Cho S.S. (2015). Association between phosphorus intake and bone health in the NHANES population. Nutr. J..

[42] Georgiou C.A., Danezis G.P., Pico Y. (2015). Elemental and Isotopic Mass Spectrometry. Advanced Mass Spectrometry for Food Safety and Quality, Comprehensive Analytical Chemistry.

