# Supplementary material for: Greek Graviera Cheese Assessment through Elemental Metabolomics—Implications for Authentication, Safety and Nutrition
Source: Molecules. 2019 Feb 14;24(4):670. doi: 10.3390/molecules24040670 (PMC6412278; doi:10.3390/molecules24040670)
Supplement: Supplementary file 1 [file molecules-24-00670-s001.pdf]

Table S1. Samples description

Sample No	Region	Sub-region	Location	Milk type
1	North Aegean	Lesvou	Andisa	sheep + goat
2	North Aegean	Lesvou	Mitilini	sheep + goat
3	North Aegean	Lesvou	Argenos	sheep + goat
4	North Aegean	Lesvou	Mitilini	sheep + goat
5	North Aegean	Lesvou	Mandamados	sheep + goat
6	North Aegean	Lesvou	Agra	sheep + goat
7	North Aegean	Lesvou	Hidira	sheep + goat
8	North Aegean	Lesvou	Mitilini	sheep + goat
9	Epirus	Artaion	Arta	sheep + goat
10	Epirus	Ioanninon	Metsovo	sheep + goat
11	Epirus	Artaion	Arta	sheep
12	Epirus	Ioanninon	Metsovo	sheep + goat
13	Epirus	Artaion	Arta	sheep + goat
14	Epirus	Ioanninon	Metsovo	sheep
15	Epirus	Artaion	Arta	sheep + goat
16	Epirus	Artaion	Arta	sheep + goat
17	Epirus	Artaion	Arta	sheep + goat
18	Crete	Rethimnis	Ag. Andreas	sheep + goat
19	Crete	Xanion	Tzitzifies Apokoronou	sheep + goat
20	Crete	Rethimnis	Kato Mallaki	sheep + goat
21	Crete	Rethimnis	Rethimno	sheep + goat
22	Crete	Rethimnis	Rethimno	sheep + goat
23	Crete	Rethimnis	Kato Mallaki	sheep + goat
24	Crete	Xanion	Sfakia	sheep + goat
25	Crete	Irakleiou	Ano Asites	sheep + goat
26	Crete	Xanion	Apokoronou	sheep + goat
27	Crete	Irakleiou	Irakleio	sheep
28	Crete	Irakleiou	Ag. Baravas Monofatsiou	sheep + goat
29	Crete	Rethimnis	Amari	sheep + goat
30	Crete	Xanion	Sfakia	sheep
31	Crete	Irakleiou	Irakleio	sheep + goat
32	Crete	Irakleiou	Irakleio	sheep + goat
33	Crete	Rethimnis	Roustika	sheep + goat
34	Crete	Rethimnis	Selia	sheep + goat
35	Crete	Rethimnis	Rethimno	sheep + goat
36	Crete	Xanion	Asi- Gonia Apokoronou	sheep + goat
37	Crete	Irakleiou	Smari Kasteliou	sheep + goat
38	Crete	Irakleiou	Kasteliana	sheep + goat

39	Crete	Rethimnis	Argiroupoli Lappaion	sheep + goat
40	Macedonia	Xalkidikis	Xalkidiki	sheep + goat
41	Macedonia	Grevenon	Grevena	cow
42	Macedonia	Grevenon	Grevena	goat
43	Macedonia	Grevenon	Grevena	goat
44	Macedonia	Grevenon	Grevena	goat
45	Macedonia	Grevenon	Grevena	cow
46	Macedonia	Grevenon	Grevena	cow
47	Macedonia	Grevenon	Grevena	goat
48	Macedonia	Serres	Serres	sheep
49	Macedonia	Serres	Serres	sheep
50	Macedonia	Serres	Serres	sheep
51	Macedonia	Serres	Serres	sheep
52	Macedonia	Kastorias	Kastoria	sheep + goat
53	South Aegean	Kikladon	Naxos	sheep + goat
54	South Aegean	Dodekanisou	Kasos	sheep + goat
55	South Aegean	Kikladon	Ios	sheep + goat
56	South Aegean	Kikladon	Paros	sheep + goat
57	South Aegean	Kikladon	Paros	sheep + goat
58	South Aegean	Kikladon	Paros	sheep + goat
59	South Aegean	Kikladon	Paros	sheep + goat
60	South Aegean	Kikladon	Paros	sheep + goat
61	South Aegean	Kikladon	Paros	sheep + goat
62	South Aegean	Kikladon	Paros	sheep + goat
63	South Aegean	Kikladon	Paros	sheep + goat
64	South Aegean	Kikladon	Paros	sheep + goat
65	South Aegean	Kikladon	Paros	sheep + goat
66	South Aegean	Kikladon	Paros	sheep + goat
67	South Aegean	Kikladon	Paros	sheep + goat
68	South Aegean	Kikladon	Naxos	sheep + goat + cow
69	South Aegean	Kikladon	Naxos	cow
70	South Aegean	Kikladon	Tinos	sheep + goat
71	South Aegean	Dodekanisou	Karpathos	goat
72	South Aegean	Dodekanisou	Karpathos	sheep + goat
73	South Aegean	Kikladon	Ios	sheep + goat
74	Peloponnese	Arkadias	Bitina	sheep + goat
75	Peloponnese	Arkadias	Tripoli	goat
76	Peloponnese	Arkadias	Tripoli	sheep + goat
77	Peloponnese	Argolidas	Nauplia	sheep
78	Peloponnese	Arkadias	Tripoli	sheep + goat

79	Peloponnese	Arkadias	Tripoli	sheep + goat
80	Peloponnese	Arkadias	Tripoli	sheep + goat
81	Peloponnese	Argolidas	Argos	sheep + goat
82	Peloponnese	Arkadias	Karitena	sheep + goat
83	Peloponnese	Korinthias	Mapsos	cow
84	Peloponnese	Arkadias	Tripoli	sheep + goat
85	Central Greece	Aitoloakarnanias	Ampelaki	sheep + goat
86	Central Greece	Aitoloakarnanias	Amfiloxia	sheep + goat
87	Central Greece	Attikis	Markopoulo	goat
88	Central Greece	Attikis	Markopoulo	sheep
89	Central Greece	Euritanias	Trixonida Karpenisi	sheep + goat
90	Central Greece	Aitoloakarnanias	Amfiloxia	sheep + goat
91	Central Greece	Aitoloakarnanias	Amfiloxia	sheep + goat
92	Central Greece	Attikis	Markopoulo	goat
93	Central Greece	Aitoloakarnanias	Amfiloxia	sheep + goat
94	Central Greece	Fthiotidas	Lamia	sheep + goat
95	Central Greece	Fthiotidas	Lamia	sheep + goat
96	Central Greece	Aitoloakarnanias	Amfiloxia	sheep + goat
97	Central Greece	Attikis	Gerakas	cow
98	Thessaly	Larisis	Larisa	sheep + goat
99	Thessaly	Trikalon	Trikala	cow
100	Thessaly	Karditsa	Mouzaki	sheep + goat
101	Thessaly	Karditsa	Mouzaki	sheep + goat
102	Thessaly	Larisis	Elassona	sheep + goat
103	Thessaly	Volou	Velestino	sheep + goat
104	Thessaly	Trikalon	Kalampaka	sheep + goat
105	Thrace	Rodopis	Komotini	Cow

Table S2. Mean value and SEM, Standard Error of the Mean (number of samples) of the elements for all milk types. The results are expressed in μg kg^{-1} except of the macro elements which are expressed in g kg^{-1}.

Analyte	Cheese Type	*Sheep+goat*		*Sheep*		*Goat*		*Cow*		*Sheep+goat+cow*	*P-value*
		Mean	SEM (n=78)	Mean	SEM (n=10)	Mean	SEM (n=8)	Mean	SEM (n=8)	One sample	
**Rare earth elements**	Ce	2.5	0.8	3	1	2.0	0.3	2.9	0.2	1.02	0.547
	Dy	0.20	0.03	0.18	0.03	0.20	0.02	0.21	0.01	0.10	0.815
	Er	0.28	0.04	0.20	0.03	0.27	0.03	0.24	0.01	0.21	0.457
	Eu	0.9	0.2	0.7	0.2	0.8	0.2	1.3	0.1	0.6	0.350
	Gd	1.1	0.6	0.6	0.1	0.60	0.05	0.67	0.05	0.23	0.327
	Ho	0.080	0.009	0.056	0.006	0.075	0.006	0.078	0.004	0.07	0.404
	La	1.5	0.5	1.7	0.6	1.3	0.2	2.0	0.2	0.5	0.577
	Lu	0.10	0.01	0.066	0.008	0.086	0.008	0.083	0.003	0.09	0.285
	Nd	1.4	0.4	1.2	0.3	1.2	0.2	1.4	0.1	0.5	0.838
	Pr	0.35	0.09	0.32	0.09	0.28	0.05	0.36	0.03	0.17	0.735
	Sc	26	4	19	1	28	2	28	1	19	0.291
	Sm	0.8	0.1	1.0	0.3	0.83	0.09	1.2	0.1	0.5	0.548
	Tb	0.19	0.02	0.23	0.06	0.24	0.02	0.18	0.01	0.19	0.560
	Tm	0.09	0.01	0.07	0.01	0.087	0.009	0.084	0.005	0.06	0.703
	Y	0.6	0.2	0.6	0.2	0.71	0.09	0.80	0.08	0.29	0.812
	Yb	0.25	0.02	0.21	0.03	0.27	0.02	0.26	0.01	0.16	0.609
**Actinides**	Th	1.8	0.1	1.4	0.1	1.7	0.1	1.52	0.05	2.04	0.146
	U	2.4	0.2	3.5	0.4	2.6	0.2	2.9	0.1	2.6	0.352
**Precious metals**	Au	3.7	0.7	4.1	0.5	4.2	0.5	4.5	0.4	3.0	0.931
	Ir	0.67	0.08	0.56	0.03	0.65	0.06	0.69	0.03	0.51	0.662
	Pd	1.7	0.3	1.5	0.3	1.6	0.2	1.73	0.08	1.00	0.743
	Pt	1.83	0.2	1.4	0.2	1.7	0.1	1.57	0.06	2.05	0.401
	Re	0.53	0.08	0.5	0.1	0.47	0.05	1.3	0.2	0.9	0.341
	Rh	4.6	0.8	6	2	4.7	0.8	5.0	0.5	5.5	0.974
	Ru	4.3	0.7	6	2	3.9	0.3	6.4	0.4	3.3	0.140
**Trace-elements**	Ag	3.5	0.3	2.5	0.2	3.8	0.3	4.1	0.2	1.9	0.118
	Al	1000	200	1700	580	1600	200	1600	200	500	0.740
	As	230	10	250	20	200	20	215	6	184	0.152
	B	40600	500	59000	8000	41000	2000	51000	2000	39000	0.216
	Ba	1000	200	800	200	900	100	1200	100	900	0.569
	Bi	16	1	60	50	15.7	0.7	14.9	0.7	10.2	0.021
	Cd	5.2	0.6	5.0	0.4	5.0	0.4	5.7	0.2	5.1	0.579

	Co	29	3	50	10	26	3	47	4	32	0.136
	Cr	520	20	610	40	510	30	600	10	460	0.047
	Cs	3.4	0.8	6	3	4.2	0.7	5.4	0.5	2.2	0.608
	Cu	730	80	690	80	550	80	750	30	500	0.257
	Fe	26000	3000	30000	2000	24000	2000	30300	600	29100	0.010
	Ga	12	1	14	4	12	1	16	1	10	0.304
	Hf	0.57	0.04	0.41	0.06	0.54	0.05	0.57	0.06	0.43	0.879
	Mn	360	40	250	20	360	30	390	20	234	0.030
	Mo	80	20	110	20	80	20	110	8	72	0.428
	Nb	1.0	0.2	0.92	0.08	0.77	0.19	1.241	0.088	0.62	0.221
	Ni	290	30	386	44	280	40	370	10	401	0.028
	Pb	28	7	15	2	27	6	26	2	6.5	0.451
	Rb	840	100	798	98	900	100	940	40	720	0.675
	Sb	5.6	0.9	4.7	0.3	4.9	0.5	5.9	0.2	3.9	0.301
	Se	60	6	99	20	43	6	79	4	49	0.016
	Si	115000	3000	140000	10000	130000	10000	135000	4000	107000	0.400
	Sn	11	5	9	2	7.2	0.9	11.5	0.9	8.63	0.560
	Sr	2700	300	2800	600	2700	400	4200	200	2000	0.010
	Ta	0.45	0.07	0.6	0.2	0.36	0.04	0.8	0.2	0.4	0.854
	Ti	57000	6000	61000	2000	49000	6000	63000	800	58600	0.001
	Tl	1.9	0.5	1.33	0.07	1.5	0.1	1.60	0.09	1.30	0.648
	V	460	50	520	50	410	30	400	20	370	0.188
	W	6	1	3.7	0.8	5.5	0.8	6.4	0.4	5.8	0.266
	Zn	31000	4000	39000	2000	25000	3000	33800	500	36200	>0.001
	Zr	4.6	0.7	5	1	4.8	0.6	5.3	0.3	2.10	0.636
**Macro-elements**	Ca	8.9	0.9	8.7	0.5	8	1	9.7	0.2	10.1	0.076
	K	0.56	0.04	0.68	0.07	0.7	0.2	0.58	0.02	0.70	0.169
	Mg	0.46	0.04	0.39	0.01	0.42	0.06	0.50	0.01	0.40	0.034
	P	6.4	0.6	7.2	0.3	5.6	0.6	7.1	0.1	6.4	0.001

*p Values lower <0.05 mean that there is a significant difference between milk types for the specific elements

Table S3. Mass of quantification, limits of Detection (LoD), limits of Quantification (LoQ) (µg kg^{-1}) and coefficient of determination.

Elemental Group	Analytes	Mass Quantification	Limit of Detection, LoD	Limit of Quantification, LoQ	R^2
Rare earth elements	Ce	140	0.017	0.051	0.9999
	Dy	164	0.016	0.048	0.9999
	Er	166	0.024	0.072	0.9999
	Eu	153	0.010	0.030	0.9999
	Gd	158	0.015	0.045	0.9999
	Ho	165	0.002	0.005	0.9999
	La	139	0.436	1.322	0.9999
	Lu	175	0.002	0.008	0.9999
	Nd	142	0.006	0.017	0.9999
	Pr	141	0.011	0.034	0.9999
	Sc	45	0.607	1.838	0.9999
	Sm	152	0.005	0.016	0.9999
	Tb	159	0.006	0.020	0.9999
	Tm	169	0.002	0.005	0.9999
	Y	89	0.006	0.018	0.9999
	Yb	174	0.008	0.024	0.9999
Actinides	Th	232	0.028	0.084	0.9999
	U	238	0.023	0.069	0.9999
Precious metals	Au	197	0.017	0.052	0.996
	Ir	193	0.001	0.003	0.9999
	Pd	106	0.041	0.123	0.9999
	Pt	195	0.006	0.019	0.9997
	Re	187	0.0003	0.0010	0.9999
	Rh	103	0.0002	0.0007	0.9999
	Ru	102	0.006	0.019	0.9999
Trace elements	Ag	107	0.020	0.061	0.9999
	Al	27	0.297	0.9	0.998
	As	75	0.07	0.20	0.999
	B	11	55	167	0.998
	Ba	138	0.059	0.177	0.97
	Bi	209	0.010	0.029	0.996

	**Cd**	111	0.269	0.816	0.994
	**Cs**	133	0.006	0.019	0.9997
	**Cr**	52	0.096	0.291	0.9999
	**Co**	59	0.015	0.046	0.9999
	**Cu**	63	20.6	62.3	0.9998
	**Fe**	57	1.8	5.3	0.996
	**Ga**	69	0.019	0.058	0.9997
	**Hf**	180	0.001	0.003	0.9999
	**Pb**	208	0.038	0.116	0.998
	**Mn**	55	0.2	0.5	0.996
	**Mo**	98	0.33	0.99	0.9991
	**Nb**	93	0.003	0.009	0.9999
	**Ni**	60	0.029	0.089	0.992
	**Rb**	65	21.2	64.2	0.9999
	**Sb**	121	0.180	0.546	0.9999
	**Se**	82	0.7	2.2	0.998
	**Si**	28	131	398	0.990
	**Sn**	118	0.040	0.122	0.9999
	**Sr**	68	2.11	6.38	0.9992
	**Ta**	181	0.003	0.009	0.9999
	**Tl**	205	0.004	0.013	0.9999
	**Ti**	47	106	320	0.997
	**V**	51	25	77	0.996
	**W**	184	0.059	0.180	0.9999
	**Zn**	66	116	352	0.999
	**Zr**	90	0.002	0.006	0.99999
**Macro elements**	**Ca**	43	26	80	0.994
	**K**	39	2.82	8.53	0.995
	**Mg**	24	64	195	0.9998
	**P**	31	119	361	0.995
